# Fertility Declines Near the End of the COVID-19 Pandemic: Evidence of the 2022 Birth Declines in Germany and Sweden

**DOI:** 10.1007/s10680-023-09689-w

**Published:** 2024-01-22

**Authors:** Martin Bujard, Gunnar Andersson

**Affiliations:** 1 Federal Institute for Population Research (BiB), Friedrich-Ebert-Allee 4, 65185 Wiesbaden, Germany; 2 Stockholm University Demography Unit (SUDA), Sociologiska Institutionen, Demografiska Avdelningen, 106 91, Stockholm, Sweden

**Keywords:** Fertility, Birth decline, COVID-19, Economic uncertainty, Vaccination, Oxford Stringency Index, Fertility plans

## Abstract

Following the onset of the COVID-19 pandemic, many countries faced short-term fertility declines in 2020–2021, a development which did not materialize in the majority of German-speaking and Nordic countries. However, more recent birth statistics show a steep fertility decline in 2022. We aim to provide empirical evidence on the unexpected birth decline in 2022 in Germany and Sweden. We rely on monthly birth statistics and present seasonally adjusted monthly Total Fertility Rates (TFR) for Germany and Sweden. We relate the nine-month lagged fertility rates to contextual developments regarding COVID-19. The seasonally adjusted monthly TFR of Germany dropped from 1.5–1.6 in 2021 to 1.4 in early 2022 and again in autumn 2022, a decline of about 10% in several months. In Sweden, the corresponding TFR dropped from about 1.7 in 2021 to 1.5–1.6 in 2022, a decline of almost 10%. There is no association of the fertility trends with changes in unemployment, infection rates, or COVID-19 deaths, but a strong association with the onset of vaccination programmes and the weakening of pandemic-related restrictions. The fertility decline in 2022 in Germany and Sweden is remarkable. Common explanations of fertility change during the pandemic do not apply. The association between the onset of mass vaccinations and subsequent fertility decline indicates that women adjusted their behaviour to get vaccinated before becoming pregnant. Fertility decreased as societies were opening up with more normalized life conditions. We provide novel information on fertility declines and the COVID-19-fertility nexus during and in the immediate aftermath of the pandemic.

## Introduction

With the onset of the COVID-19 pandemic, many scholars expected the pandemic to have a negative impact on fertility developments (Aassve et al., 2020; Berrington et al., 2022a). Two main mechanisms were assumed to be at play: the impact of the health crisis and the impact of pandemic-induced economic uncertainties on fertility plans. Current knowledge on the influence of the COVID-19 pandemic on fertility patterns is mixed and findings vary between countries and the timing of infection waves, shutdown policies, and pre-existing fertility changes. For many high-income countries, monthly birth counts declined between November 2020 and January 2021, i.e. nine months after the onset of the pandemic during March–May 2020. The declines were particularly strong in southern Europe (Aassve et al., 2021; Sobotka et al., 2021, 2023) and occurred with considerable within-country heterogeneity (Arpino, Luppi, & Rosina, 2021). In Spain, the monthly Total Fertility Rate (TFR) declined with some 20% to a level below 1.0 in December 2022 (Cozzani et al., 2022), the sharpest drop observed in Europe (Sobotka et al., 2021, 2023). Fertility declines during the transition from 2020 to 2021 were also observed for Japan (Ghaznavi et al., 2022), the USA (Gromski et al., 2020; Hamilton, Martin, & Osterman, 2021), and the UK (Berrington et al., 2022b).

However, in Nordic and some German-speaking countries the fertility patterns were somewhat different. In Sweden (Neyer et al., 2022), Norway (Lappegård et al., 2022), Finland (Nisén et al., 2022), and Germany (Pötzsch, 2021), there was no visible fertility decline in late 2020 or early 2021. In contrast, these countries even experienced minor increases in their monthly fertility rates in early 2021 as well as during the autumn of the same year. Explanations to the positive fertility trends during the course of the COVID-19 pandemic range from the less severe mortality impacts than in many other contexts to the buffering role of protective social policies and swiftly introduced economic-support programmes during the early phases of the pandemic. Analyses based on 17 European countries reveal that the role of perceived uncertainty regarding job markets and household finances reduced fertility in other contexts at the very onset of the pandemic (Tavares, Azevedo, & Arpino, 2022).

However, in the final stage of the pandemic, monthly fertility data from Sweden and Germany show a strong fertility decline in early 2022, with about 10–15% less births, respectively, than what was observed during the same period the previous year. This poses questions on the role of previously suggested mechanisms for pandemic-related fertility change, such as the role of health-related or economic-centred factors in recent fertility change. It also brings factors related to the perceived cessation of the pandemic to our attention, as reflected in the onset of broad-based vaccination programmes directed at the population at reproductive and economically active ages. The first vaccines were made available already at the very end of 2020 and were initially aimed at specific groups of employees in the healthcare system, at older people, and those with an underlying health condition. The vaccination programmes were later expanded to cover the general population and in most European countries vaccination intensities reached its peak during the spring and summer of 2021 (Antonini et al., 2022). If there is an impact of these interventions on childbearing behaviour, it should be observed from the turn of 2021–2022 and onwards.

The current study aims to describe the fertility-trend change that occurred in Germany and Sweden during early 2022 by presenting statistics on monthly live births and seasonally adjusted monthly TFR prior to and during the course of the pandemic. Further, we compare our monthly fertility indicators with contextually relevant developments for a few pandemic-related factors, including the onset of broad-based vaccination programmes in the two countries we study. We expect our contribution to be helpful for future research when developing new hypotheses on the different factors that may contribute to family-related change as societies exit from their pandemic-driven circumstances.

## Four Relevant Influences of the COVID-19 Pandemic on Childbearing Behaviour

The most obvious influence of the COVID-19 pandemic on fertility trends is through different factors that relate to the *health crisis* as such. For example, evidence from previous global pandemics indicates that fertility declined after the H1N1 “Spanish Flu” of 1918–19 in Britain (Reid, 2005), Japan (Chandra & Yu, 2015), and the USA (Chandra et al., 2018). The fertility decline in US cities was about 20% nine months after the peak of that pandemic but recovered where public health interventions were implemented (Wagner et al., 2020). However, these historical experiences cannot be transferred directly to the contemporary situation as healthcare and economic welfare systems are now much more developed than a century ago. Also, the Spanish Flu mainly had an impact on persons at childbearing and economically active ages (Reid, 2005) while COVID-19 mortality and morbidity have had the strongest impact on people at more advanced ages (Bonanad et al., 2020; Kolk et al., 2022). However, the healthcare system was partly overstrained also during the COVID-19 pandemic, resulting in reduced support in patient fertility care for assisted reproductive procedures and for birth clinics in general (DSouza et al., 2022).

The impact of *economic crises* as triggered by the global pandemic, and the perception of economic uncertainty during the course of the pandemic, is another mechanism that could relate to reduced fertility intentions and childbearing behaviour. A negative relation between employment instability, aggregate unemployment, and fertility is well known (Adsera, 2011; Albeitawi et al., 2022). The Great Recession in Europe during 2007–2008 was negatively related to subsequent fertility trends, however, with considerable differences by age, birth parity, and regions in Europe (Goldstein et al., 2013). Higher levels of unemployment at the regional level seem to be negatively related to fertility trends (Matysiak et al., 2021) and cohort fertility (Bujard & Scheller, 2017). However, subjective indicators such as individuals’ perceptions of economic uncertainty may often matter more for couples’ fertility decisions than their actual economic situation (Comolli et al., 2021; Kreyenfeld, 2016; Vignoli et al., 2020).

While the health crisis and different aspects of pandemic-induced economic uncertainty are expected to bring negative influences on fertility, there could also be a positive influence from the life circumstances during the pandemic that could be labelled a *cocooning effect*. There was huge heterogeneity in families’ experiences and life circumstances while social distancing policies and other interventions were in effect in people’s lives during the pandemic, but sometimes these may have led to a more family-oriented life situation (Ahmed et al., 2020). Increased time by parents to care for their children and, in the case of Germany, for home-schooling were often challenging but sometimes also provided opportunities for more value-based behaviour (Szabo et al., 2020). Partners may have had more time to talk about their fertility plans and perhaps more opportunity for sexual intercourse (Berrington et al., 2022b). An increased attention to the value of children (Hoffman & Hoffman, 1973) and more time for couple interaction may for some have resulted in stronger childbearing intentions.

The mechanisms behind the onset of large-scale *vaccination programmes* on fertility have not yet been analysed. These programmes mark the ending of the pervasiveness of the global pandemic on people’s lives and the life situation that had prevailed during the pandemic. They signalled a return to the less family- and home-centred life situation that prevailed before the onset of the pandemic. Another factor could be that any perceived fear that the COVID-19 vaccine had a negative impact on women and men’s fecundity, which in some cases was labelled a “major cause of vaccine hesitancy” (Diaz et al., 2022), affected childbearing considerations. Further, the official recommendation to get vaccinated during pregnancy was initially hesitant but later changed during the course of vaccination programmes. Also, the vaccination uptake for pregnant women was lower than for the general population (Januszek et al., 2021). In some cases, unvaccinated women could have postponed their fertility plans to the time after having got vaccinated. We expect different mechanisms to be at play in different phases of the pandemic, which can be labelled as the quarantine phase, and the early and large-scale immunization phases. The WHO declared the end of Covid‐19 as a global health emergency in May 2023, and we consider the phase where most people have become immunized as the aftermath of the pandemic.

## Data and Methods

Monthly data on live births in Germany during 2000–2021 were drawn from the German birth register (Statistisches Bundesamt, 2023c). For 2022 and January 2023, we use preliminary data on live births, by birth month, which differ somewhat from statistically recorded notifications of births (Statistisches Bundesamt, 2023b). We estimated monthly Total Fertility Rates (TFR) based on annual TFRs, monthly fertility data, and monthly female population exposures (Jdanov et al., 2022). The estimation method is based on the calculation of the General Fertility Rate (GFR), based on the linear interpolation of female population aged 15–44, and the annual relation between the GFR and TFR. The monthly GFR is estimated as follows:

1 $${\mathrm{GFR}}_{m, y} = \frac{{B}_{m,y }}{{\mathrm{FP}}_{m, y}(\mathrm{15,44})}$$

The GFR displays monthly (or yearly) birth rates as the fraction between live births (*B*) and the female population (FP) in reproductive age (15–44) in a specific period. Data of monthly live births are available from the German birth register (Statistisches Bundesamt, 2023c); data on the female population aged 15–44 is calculated by linear interpolation of yearly data for December 31st each year from German population statistics (Statistisches Bundesamt, 2023a). Since the age structure of the female population aged 15–44 is relatively stable over the course of a year, we assume that the relation between the GFR and the TFR is stable, too. Therefore, we calculate the annual relation between the two measures (*r*) as follows:

2 $${r}_{y} = \frac{{\mathrm{TFR}}_{y }}{{\mathrm{GFR}}_{ y}}$$

We calculate monthly r’s by interpolation between the annual *r* values and monthly TFRs as follows:

3 $${\mathrm{TFR}}_{m} = {\mathrm{GFR}}_{m}* {r}_{m}$$

Since monthly changes in the population exposure are rather small and estimations for monthly TFRs are strongly influenced by seasonal patterns of fertility fluctuation, we adjusted for seasonal effects by calculating a seasonal adjustment factor based on the average seasonal patterns between 2000 and 2020:

4 $${s}_{m} = \frac{{\mathrm{mean TFR}}_{m}2000-2020}{(\mathrm{mean }{\mathrm{TFR}}_{y}2000-2020) /12}$$

The seasonal adjustment factor s is calculated by the relation between the mean of the TFR for a specific month for the years 2000–2020 and the mean of all months in the same period.

5 $${\mathrm{TFR}}_{m}^{s} = \frac{{\mathrm{TFR}}_{m}}{{s}_{m}}$$

The seasonal adjusted TFR for a specific month (${\mathrm{TFR}}_{m}^{s})$ is calculated by the fraction between the monthly TFR and its seasonal adjustment factor.

Swedish data on live births and women at reproductive ages stem from the country’s population register and are available at Statistics Sweden (Statistics Sweden, 2023). The data are updated on a monthly basis and provided for women in Sweden and births by age of mother by the accuracy of single-year age groups. Statistics Sweden also produces time series of monthly TFRs, including seasonally adjusted series of such fertility rates. The TFRs are calculated by means of conventional summaries of the age-specific fertility rates for each month under observation. Statistics Sweden’s seasonality weights are updated on a regular basis as seasonality patterns in Sweden have changed somewhat during recent decades (Dahlberg & Andersson, 2018). They are currently based on a rolling average of the last five years of monthly seasonality observations (Lundkvist, 2023).

In our presentation, we also relate the developments in birth statistics with monthly data on a few relevant contextual indicators which we observe nine months before the childbirths we cover. With regard to the health crisis, we consider the number of COVID-19 related deaths and the seven-day infection incidence in Sweden and Germany (Robert-Koch-Institute, 2022a, 2023). Regarding economic factors, we consider the monthly unemployment rates in Germany and Sweden (Statistics Sweden, 2022) and, for Germany, the number of employees taking short-work social-security benefits (“Kurzarbeit”). The latter programme helped employees not become unemployed and can be seen as an indicator of the degree of job insecurity during the course of the pandemic (Bundesagentur für Arbeit, 2022, 2023). As a third contextual factor, we consider the vaccination programmes and its interventions with a first, second, and third vaccination event in Germany (Robert-Koch-Institute, 2022b, 2023) and a first and second vaccination event in Sweden (Public Health Agency of Sweden, 2022b). Our fourth contextual factor is the strictness of applied pandemic-related restrictions, which varies over time and between countries; we use the Oxford Stringency Index as our indicator for this outcome (Hale et al., 2021).

## Results

### Fertility Developments in Relation to Previous Trends: Monthly TFRs in Germany and Sweden in the Twenty-first Century

**Fig. 1:**
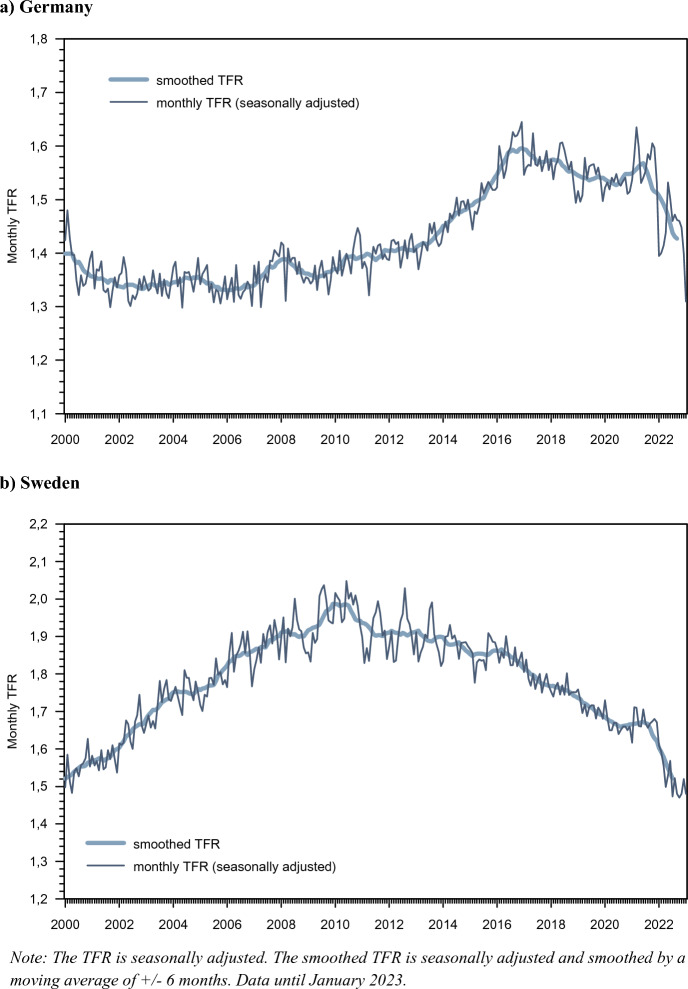
Monthly Total Fertility Rate (TFR) for Germany and Sweden, 1/2000–1/2023. **a** Germany. **b** Sweden. *Source* Own calculations based on Germany’s birth statistics and current updating of population statistics. Own smoothing of monthly TFR data produced by Statistics Sweden

Between the years 2000 and 2014, Germany’s TFR was constantly hovering at a level between 1.3 and 1.5 children per woman (Fig. 1). In contrast, from 2015 to 2021 it was on an upward trend, to a TFR level close to 1.7. A peak occurred during the COVID-19 pandemic in March and October 2021 with a TFR level clearly above 1.60. However, in the first months of 2022 there was an abrupt decline in birth rates so that the TFR reached a level of 1.39 in January 2022, which slightly increased to 1.44 in April. In May and June fertility rates recovered, the TFR rebounded to 1.5. However, this recovery was only short-lived and the TFR decreased again to 1.45 and 1.39 in November and December 2022 and to 1.31 in January 2023.

The TFR trends in Sweden during the first two decades of the new century were markedly different from those in Germany (Fig. 1): Sweden’s TFRs first increased during the first decade of the twenty-first century, to a level of about 2.0 in much of 2010, then declined during its second decade. The initial increase amounted to a recuperation of the depressed fertility and postponed childbearing that occurred during the 1990s. The latter decline occurred in tandem with similar fertility declines in other countries in Northern and Western Europe as well as in Anglo-Saxon countries. It was driven by declines in first-birth rates of women and men in couples (Ohlsson-Wijk & Andersson, 2022). However, the fertility patterns during the COVID-19 pandemic were largely similar to those in Germany. During the pandemic, the previous fertility decline was reversed and Sweden’s TFR hovered at a seasonally adjusted level of 1.65–1.71. As in Germany, it subsequently showed a drastic decline in its monthly TFR when the pandemic came to a halt: During the first months of 2022, the Swedish TFR fell to a markedly depressed level and stabilized during the spring at a level of close to 1.5, which is close to the record-low of Swedish TFRs. The seasonally adjusted TFR remained at that level for the rest of the year and in early 2023.

### Changes in the Number of Live Births Per Month During the Course of the Covid-19 Pandemic

In contrast to many other European countries, Germany experienced no birth decline in the first months of 2021. There was even a small increase of about 2.9% in the total number of births in 2021 as compared to the previous year; the increase was particularly pronounced during February and March and during October to December 2021. In contrast, there is a subsequent decline of 7.1% in the number of births in 2022. During January to April 2022, this decline was between 6.6 and 8.5% as compared to the 5-year average of 2016–2020 (Table 1). In May and June 2022, the number of births increased somewhat. However, during July to November the decline was between 5 and 8% as compared to the 5-year average of 2016–2020.

**Table 1:** Trends in the number of births in Germany, by month in 2021–22. *Source* Own calculations based on Germany’s birth statistic, 2018–21: Statistisches Bundesamt (2023b, 2023c)

	Live births 2021	Live births 2022	Change 2021 vs. 2020 (in %)	Change 2022 vs. 2021 (in %)	Change 2021 vs. mean 2016–20 (in %)	Change 2022 vs. mean 2016–20 (in %)
January	64,325	58,406	0.96	− 9.20	0.94	− 8.35
February	60,595	53,727	3.23	− 11.33	3.26	− 8.45
March	67,060	58,135	7.76	− 13.31	6.74	− 7.47
April	63,601	57,450	3.86	− 9.67	3.42	− 6.58
May	64,748	65,084	0.07	0.52	− 1.73	− 1.22
June	65,985	64,664	− 0.19	− 2.00	− 1.23	− 3.21
July	72,267	68,385	1.70	− 5.37	− 0.23	− 5.59
August	72,542	67,948	4.09	− 6.33	1.49	− 4.93
September	71,362	66,935	2.74	− 6.20	1.54	− 4.76
October	68,822	63,375	4.25	− 7.91	2.71	− 5.42
November	62,895	57,829	5.70	− 8.05	2.92	− 5.37
December	61,290	56,918	0.95	− 7.13	− 0.23	− 7.34

The patterns of monthly increases and subsequent declines in the number of births in Sweden in 2021 and 2022 were very similar to those observed for Germany. Except for a decline in births in January 2021, the number of births in that year was slightly higher than in 2020 and only slightly lower than for the preceding 5-year average of observations. The declines in the number of live births in early 2022 were also remarkable, showing a subsequent decline of 8.3% in the number of births in 2022. During the course of the spring, the decline intensified and led to a reduction in the number of births in April, July, September, October, and November of more than 10% in relation to those of the preceding 5-year averages of observations (Table 2).

**Table 2:** Trends in the number of births in Sweden, by month in 2021–22. *Source* Calculations based on data from Statistics Sweden (2023)

	Live births 2021	Live births 2022	Change 2021 vs. 2020 (in %)	Change 2022 vs. 2021 (in %)	Change 2021 vs. mean 2016–20 (in %)	Change 2022 vs. mean 2016–20 (in %)
January	9 071	8 917	− 6,26	− 1,70	− 5,20	− 6,81
February	8 989	8 546	0,39	− 4,93	− 0,02	− 4,95
March	10 067	9 194	4,36	− 8,67	1,12	− 7,65
April	9 823	8 816	1,13	− 10,25	− 1,92	− 11,98
May	10 322	9 580	− 0,83	− 7,19	− 1,72	− 8,78
June	10 216	9 531	3,43	− 6,71	1,75	− 5,07
July	10 325	9 232	2,04	− 10,59	− 0,69	− 11,21
August	10 082	9 369	1,24	− 7,07	− 0,94	− 7,94
September	9 419	8 374	1,03	− 11,09	− 1,60	− 12,52
October	9 316	8 147	1,55	− 12,55	− 0,20	− 12,72
November	8 492	7 506	3,64	− 11,61	0,40	− 11,26
December	8 141	7 522	1,34	− 7,60	− 1,22	− 8,73

### Fertility Change in the Context of Health Crisis, Economic Hardship, Vaccination Programmes, and Pandemic-Related Restrictions

**Fig. 2:**
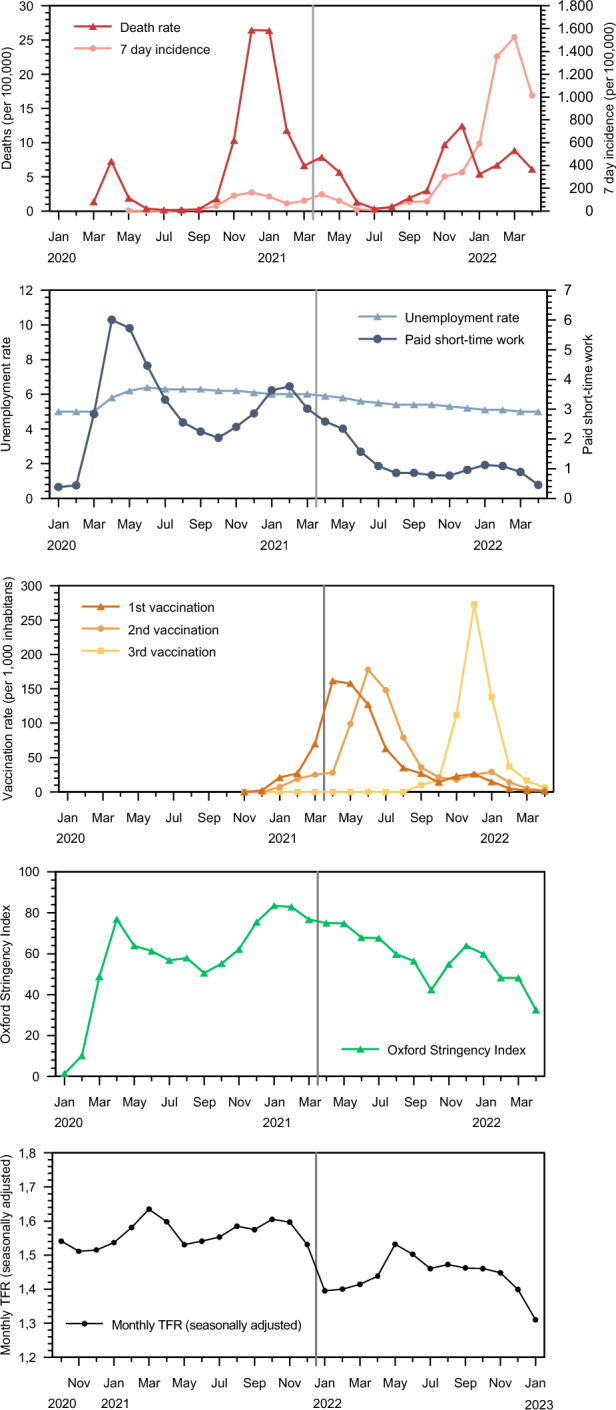
COVID-19 measures, employment, and vaccinations in 1/2020–4/2022 in Germany and nine months lagged TFRs for 10/2020–1/2023. * Source* Own diagram, data on deaths and incidences based on Robert-Koch-Institute (2022a), (2023) data on short-time work benefit and unemployment based on Bundesagentur für Arbeit (2022), (2023), data on vaccinations based on Robert-Koch-Institute (2022b), Oxford Stringency Index (Hale et al., 2021), data on monthly TFR see Fig. 1

**Fig. 3:**
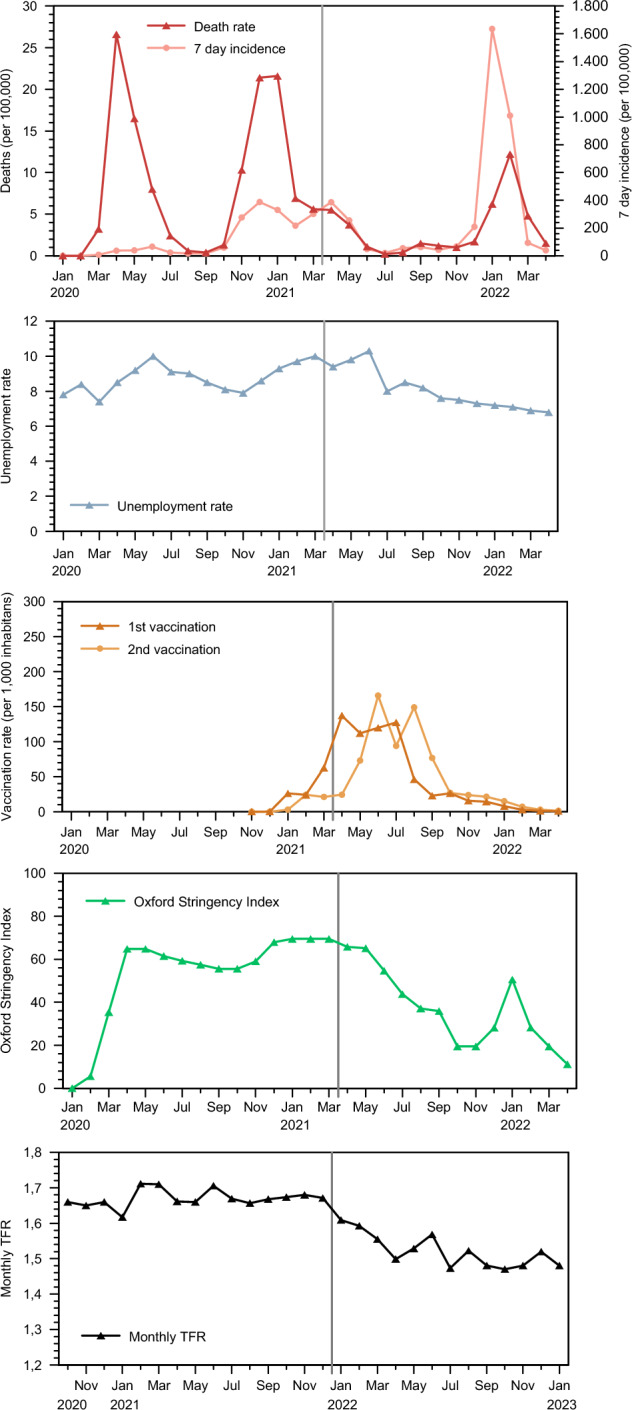
COVID-19 measures, unemployment, and vaccinations in 1/2020–4/2022 in Sweden and nine months lagged TFRs for 10/2020–1/2023. *Source* Own diagram, 7-day incidence, and vaccinations are calculated based on data available at Ritchie et al. (2022), data on deaths available from the Swedish National Board of Health and Welfare (2022), data on unemployment available from Statistics Sweden’s Labour-force Surveys (Statistics Sweden, 2023), Oxford Stringency Index (Hale et al., 2021), data on monthly TFR see Fig. 1

In this section, we relate the monthly fertility patterns in Germany and Sweden during and in the immediate aftermath of the pandemic to a few crucial contextual developments which we display in relation to birth data that are adjusted nine months to the time of conception of the children born (cf. Fig. 2 for Germany; Fig. 3 for Sweden). The monthly contextual developments are covered by data on COVID-19 mortality, 7-day incidence rates of COVID-19, unemployment rates, utilization of the German short-time work programme (‘Kurzarbeit’), vaccination events, and the Oxford Stringency Index. In Germany, a first modest increase of COVID-19 related mortality occurred in April 2020 while a more substantial peak in death rates occurred during December 2020–January 2021. There was also a third wave of COVID-19 deaths towards the end of 2021. In Sweden, the first two mortality peaks occurred at rather similar times: during April–May 2020 and November–December 2020–January 2021, but with a much stronger first wave of COVID-19 mortality than in Germany. In contrast, towards the end of 2021 Sweden had very low COVID-19 mortality. In January 2021 and September/October 2021, which is nine months after the two peaks of COVID‐19 mortality, we observe no fertility declines. Actually, nine months before the fertility decline in early 2022, i.e. during April–July 2021, the number of COVID-19 deaths and the incidences of COVID-19 infections were fairly low in both countries.

Further, in Germany the unemployment rate was increasing slightly in April–May 2020. Germany’s short-time work programme (‘Kurzarbeit’) also reached its peak with more than 6 million employees in paid short-time work during these two months. In January and February 2021, which is nine months after this peak in labour market volatility, there was no fertility decline. A similar lack of a clear relationship between unemployment rates and subsequent fertility is observed for Sweden. Swedish unemployment peaked with elevated unemployment rates in January–June 2021 and with no clear association with birth outcomes nine months later during October 2021–March 2022, which corresponds to the time both before and after the fertility decline of interest. It also corresponds to periods both before and during the process of mass vaccinations in Sweden. Later on, unemployment levels declined as Swedish society opened up with a labour force of vaccinated workers.

In contrast, there is a clear temporal correlation between the onset of vaccination programmes and fertility developments nine months later. In Germany as well as in Sweden, the vaccination campaigns with mass enrolments for a first vaccination reached its peak in April, May, and June 2021, followed by a wave of second vaccinations with its peak between May and August the same year. (Two vaccinations were considered being fully vaccinated.) The implementations of these programmes in both Germany and Sweden are concurrent with a distinct change in fertility levels in January–March 2022 that is exactly nine months later. After a tentative and temporary recovery in Germany, the fertility rates remained at reduced levels in the autumn of 2022, which corresponds to the timing of third vaccinations nine months earlier in December 2021. In Sweden, fertility rates remained depressed throughout the entire 2022.

In addition, there is also a clear association between the opening of societies as measured by the Oxford Stringency Index and the fertility development nine months later. As vaccination programmes begun, societies could open up and the Oxford Index begun a trend of decreasing levels of Stringency, for both Germany and Sweden. For both countries, the Stringency Index displayed its peak levels immediately before the onset of declining conception rates. As a matter of fact, the association between the trend changes in the Stringency Index and in conceptions that lead to live births appears robust.

More in-depth analyses based on Swedish micro data reveal that the decline in fertility during 2022 mainly was confined to groups that temporarily had shown elevated fertility levels during the preceding year in 2021 (Ohlsson-Wijk & Andersson, 2023). This mainly holds for second and third births (Lundkvist, 2022), and for women in metropolitan areas, at peak childbearing ages, and with high levels of income (Ohlsson-Wijk & Andersson, 2023). The trend change thus indicates a reversal of the situation during the pandemic itself when parents of one and two children sometimes took the opportunity to speed up their childbearing with the arrival of a next, already planned child (Neyer et al., 2022). To speed up continued childbearing during a situation when many parents were confined to their homes could sometimes be a rational use of parenting time. This would amount to a version of the cocooning effect in childbearing behaviour that we discussed earlier, and which ended when societies opened up again. However, aggregate fertility rates in 2022 were lower than their pre‐pandemic levels, which suggest that additional behavioural factors might have been at play. For Sweden, a resumption of the pre-pandemic trend of declining first-birth rates was at play (Ohlsson-Wijk & Andersson 2023). Taken together, the observations of parity-specific fertility changes during 2021–2022 (not shown here) suggest that a large part of the post-pandemic fertility change can be ascribed to behavioural changes that were in reaction to societies opening up to less home-centred life circumstances than those prevailing during the pandemic.

However, there could initially also have been a more direct role of the vaccination programmes as such on childbearing considerations. During the course of vaccination programmes, in Germany recommendations for pregnant women changed in the light of increasing evidence of the security of vaccines for pregnant women. In January 2021, there was no official recommendation for the vaccination of pregnant women by the permanent vaccination commission of Germany (Robert-Koch-Institute, 2021a). It lasted until September 23^rd^ the same year when this commission gave an explicit recommendation for pregnant women to get vaccinated against COVID-19 and labelled them as an “explicit target group” (Robert-Koch-Institute, 2021b). The lack of initial recommendations could have propelled some prospective mothers to postpone childbearing until after getting a vaccination for themselves. In Sweden, pregnant women were rarely singled out as an explicit category when prioritizing population sub-groups for vaccination access. However, from May 2021 onwards they were explicitly offered vaccinations to the same extent as the general population of Sweden (Public Health Agency of Sweden, 2023). Nevertheless, in October 2022, the Public Health Agency of Sweden felt compelled to explicitly provide positive vaccination advice for pregnant women as a specific population sub-group (Public Health Agency of Sweden, 2022a).

## Discussion

This study has demonstrated a remarkably strong and very sudden drop in fertility in Germany and Sweden in the first months of 2022 followed by low fertility rates also in late 2022 and early 2023. The number of live births dropped by some 10% in the beginning of 2022 in Germany and close to 10% in Sweden. The fertility decline was very different from the slower pace of change that usually characterize fertility developments. It happened as societies were to open up after two years of COVID-19 related restrictions in people’s lives. More precisely, the fertility decline occurred some nine months after the implementation of broad-based vaccination programmes for the general population in Germany and Sweden. In the wake of these interventions, the seasonally adjusted monthly TFR of Germany dropped from a level during 2016–2021 of 1.5–1.6 children per woman to a lowest-low fertility level of 1.4. In Sweden, the decline occurred from a slightly higher level of departure, of 1.6–1.7 during 2018–2021 to a level close to 1.5 in 2022, but with a similar direction and magnitude. These declines are remarkable for two reasons: First, Germany and Sweden are countries that experienced no fertility decline during the course of the pandemic itself, in 2020 and 2021. Second, both countries reached fertility levels that were lower than what had been experienced for many years.

Other well-known explanations of fertility change during the course of the pandemic, such as the impact of health-related and economic factors seem not to be associated with the timing of fertility decline in 2022. Based on the descriptive associations presented in this study, we interpret the post-pandemic change in childbearing behaviour as a reaction to the changes in life circumstances that were anticipated as societies were to open up to non-pandemic conditions. In some cases, there may have been a more direct effect of the vaccination programme as such, as some prospective parents may have postponed a decision to have another child until after securing a vaccination for themselves. These interpretations also have implications for our expectations for future fertility developments. If reactions to the opening up of societies and the postponement of childbearing in anticipation of vaccination access were the main driving forces of the 2022 fertility decline, we would soon expect a return to pre-pandemic fertility trends. For Germany, these trends may be more positive than for Sweden. In Sweden, as in all Nordic countries (Hellstrand et al., 2021), the pre-pandemic trends were decisively pointing in a negative direction.

However, there are also arguments against the expectation of a straightforward return to pre-pandemic fertility trends. First, many families also had difficult times during the period of pandemic-related restrictions. Parental stress increased (Calvano et al., 2022), the relative well-being of parents decreased (Huebener et al., 2021), and for children and adolescents in European countries a remarkable increase in depression (Ludwig-Walz et al., 2022) and anxiety (Ludwig-Walz et al., 2023) occurred. These experiences of restrictive measures at the expense of families could all contribute to future depressed fertility. Second, the impact of war in Europe and high inflation could contribute to increasing feelings of uncertainty (Comolli et al., 2021; Kreyenfeld, 2016; Vignoli et al., 2020), which also might lead to lower fertility in the near future.

There are several limitations of our study. The most recent monthly data for Germany are still preliminary and may be corrected later. However, such corrections will not change the extent of fertility decline in any substantial manner. The estimation of monthly TFRs and the seasonal adjustments that we apply also depend on assumptions of seasonal patterns during previous comparison periods that may later be challenged. The biggest limitation is that our interpretations are based on descriptive associations that do not account for the many individual-level characteristics and other contextual factors that may also be at play. More in-depth research based on individual-level data will provide better insight into the nature of the observed fertility decline in Germany as well as in Sweden, when such data are available.

Our study still provides valuable data and insight on a new and entirely unanticipated fertility development in the context of the COVID-19 pandemic. It remains to be seen whether these developments are of a short-term nature and if and how fast fertility trends in Germany and Sweden will return to their pre-pandemic patterns, which for Germany was running in an upward direction and in Sweden in a downward direction.

## Figures and Tables

**Fig. 4:**
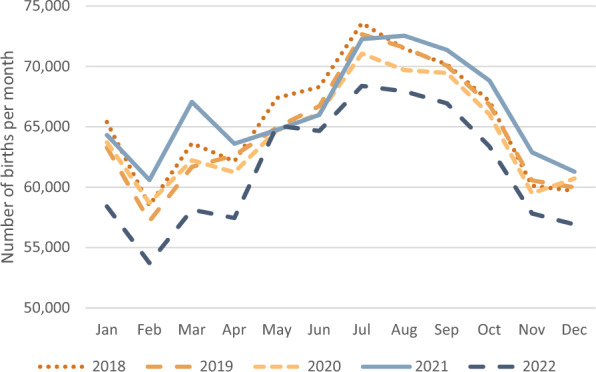
Trends in the number of births in Germany, by month in 2018–2022. *Source* Own calculations based on Germany’s birth statistic, 2018–22: Statistisches Bundesamt (2023c), Statistisches Bundesamt (2023b)

**Fig. 5:**
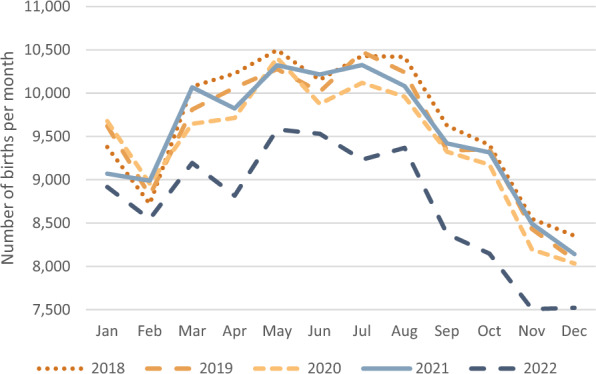
Trends in the number of births in Sweden, by month in 2018–2022. *Source* Statistics Sweden (2023), Statistikdatabasen

## Appendix

See Figs. 4, 5.

## References

[CR1] Aassve, A., Cavalli, N., Mencarini, L., Plach, S.,& Sanders, S. (2021). Early assessment of the relationship between the COVID-19 pandemic and births in high-income countries. *Proceedings of the National Academy of Sciences of the United States of America (PNAS), 118*(36). 10.1073/pnas.2105709118 10.1073/pnas.2105709118 PMC8433569 34462356

[CR2] Aassve, A., Cavalli, N., Mencarini, L., Plach, S., & Livi Bacci, M. (2020). The COVID-19 pandemic and human fertility: Birth trends in response to the pandemic will vary according to socioeconomic conditions. *Science,**369*(6502), 370–371. 10.1126/science.abc9520 32703862 10.1126/science.abc9520

[CR3] Adsera, A. (2011). Where are the babies? Labor market conditions and fertility in Europe. *European Journal of Population,**27*(1), 1–32. 10.1007/s10680-010-9222-x 23580794 10.1007/s10680-010-9222-x PMC3620442

[CR4] Ahmed, D., Buheji, M., & Fardan, S. M. (2020). Re-emphasising the future family role in ‘care economy’ as a result of Covid-19 pandemic spillovers. *American Journal of Economics,**10*(6), 332–338.

[CR5] Albeitawi, S., Al-Alami, Z., Khamaiseh, K., Al Mehaisen, L., Khamees, A., & Hamadneh, J. (2022). Conception preferences during COVID-19 pandemic lockdowns. *Behavioral Sciences*. 10.3390/bs12050144 10.3390/bs12050144 PMC9137999 35621441

[CR6] Antonini, M., Eid, M. A., Falkenbach, M., Rosenbluth, S. T., Prieto, P. A., Brammli-Greenberg, S., & Paolucci, F. (2022). An analysis of the COVID-19 vaccination campaigns in France, Israel, Italy and Spain and their impact on health and economic outcomes. *Health Policy and Technology,**11*(2), 100594. 10.1016/j.hlpt.2021.100594 34976711 10.1016/j.hlpt.2021.100594 PMC8702636

[CR7] Arpino, B., Luppi, F., & Rosina, A. (2021). Regional trends in births during the COVID-19 crisis in France, Germany, Italy, and Spain: Preprint. Advance online publication. 10.31235/osf.io/mnwh8

[CR8] Berrington, A., Ellison, J., Kuang, B., Vasireddy, S., & Kulu, H. (2022b). What is the likely impact of Covid-19 on fertility in the UK? ESRC Centre for Population Change Policy Briefing. (66). Retrieved from https://eprints.soton.ac.uk/454339/

[CR9] Berrington, A., Ellison, J., Kuang, B., Vasireddy, S., & Kulu, H. (2022a). Scenario-based fertility projections incorporating impacts of COVID-19. *Population, Space and Place*. 10.1002/psp.2546

[CR10] Bonanad, C., García-Blas, S., Tarazona-Santabalbina, F., Sanchis, J., Bertomeu-González, V., Fácila, L., & Cordero, A. (2020). The effect of age on mortality in patients with COVID-19: A meta-analysis with 611,583 subjects. *Journal of the American Medical Directors Association,**21*(7), 915–918. 10.1016/j.jamda.2020.05.045 32674819 10.1016/j.jamda.2020.05.045 PMC7247470

[CR11] Bujard, M., & Scheller, M. (2017). Impact of regional factors on cohort fertility: New estimations at the district level in Germany. *Comparative Population Studies,**42*, 55–88. 10.12765/CPOS-2017-07EN

[CR12] Bundesagentur für Arbeit. (2022). Monatsbericht zum Arbeits- und Ausbildungsmarkt: Juni 2022 (Blickpunkt Arbeitsmarkt). Nürnberg

[CR13] Bundesagentur für Arbeit. (2023). Monatsbericht zum Arbeits- und Ausbildungsmarkt: Dezember und Jahr 2022 (Blickpunkt Arbeitsmarkt). Nürnberg

[CR14] Calvano, C., Engelke, L., Di Bella, J., Kindermann, J., Renneberg, B., & Winter, S. M. (2022). Families in the COVID-19 pandemic: Parental stress, parent mental health and the occurrence of adverse childhood experiences—results of a representative survey in Germany. *European Child & Adolescent Psychiatry,**31*(7), 1–13. 10.1007/s00787-021-01739-0 10.1007/s00787-021-01739-0 PMC7917379 33646416

[CR15] Chandra, S., Christensen, J., Mamelund, S.-E., & Paneth, N. (2018). Short-term birth sequelae of the 1918–1920 influenza pandemic in the United States: State-level analysis. *American Journal of Epidemiology,**187*(12), 2585–2595. 10.1093/aje/kwy153 30059982 10.1093/aje/kwy153 PMC7314232

[CR16] Chandra, S., & Yu, Y.-L. (2015). The 1918 influenza pandemic and subsequent birth deficit in Japan. *Demographic Research,**33*(11), 313–326. 10.4054/DemRes.2015.33.11

[CR17] Comolli, C. L., Neyer, G., Andersson, G., Dommermuth, L., Fallesen, P., Jalovaara, M., & Lappegård, T. (2021). Beyond the economic gaze: Childbearing during and after recessions in the Nordic countries. *European Journal of Population,**37*(2), 473–520. 10.1007/s10680-020-09570-0 33230356 10.1007/s10680-020-09570-0 PMC7676408

[CR18] Cozzani, M., Fallesen, P., Passaretta, G., Härkönen, J., & Bernadi, F. (2022). The consequences of the COVID-19 pandemic for fertility and birth outcomes: Evidence from Spanish birth registers. *Stockholm Research Reports in Demography*. 10.1111/padr.12536

[CR19] Dahlberg, J., & Andersson, G. (2018). Changing seasonal variation in births by sociodemographic factors: A population-based register study. *Human Reproduction Open,**4*(15), 1–8. 10.1093/hropen/hoy015 PMC6276686 30895256

[CR20] Diaz, P., Zizzo, J., Balaji, N. C., Reddy, R., Khodamoradi, K., Ory, J., & Ramasamy, R. (2022). Fear about adverse effect on fertility is a major cause of COVID-19 vaccine hesitancy in the United States. *Andrologia,**54*(4), e14361. 10.1111/and.14361 34970749 10.1111/and.14361

[CR21] Dsouza, K. N., Orellana, M., Ainsworth, A. J., Cummings, G., Riggan, K. A., Shenoy, C. C., & Allyse, M. A. (2022). Impact of the COVID-19 pandemic on patient fertility care. *Journal of Patient Experience,**9*, 1–7. 10.1177/23743735221098255 PMC9083039 35548663

[CR22] Ghaznavi, C., Kawashima, T., Tanoue, Y., Yoneoka, D., Makiyama, K., Sakamoto, H., & Nomura, S. (2022). Changes in marriage, divorce and births during the COVID-19 pandemic in Japan. *BMJ Global Health*. 10.1136/bmjgh-2021-007866 10.1136/bmjgh-2021-007866 PMC9108437 35569835

[CR23] Goldstein, J. R., Kreyenfeld, M., Jasilioniene, A., & Karaman Örsal, D. D. (2013). Fertility reactions to the “great recession” in Europe: Recent evidence from order-specific data. *Demographic Research,**29*(4), 85–104. 10.4054/DemRes.2013.29.4

[CR24] Gromski, P. S., Smith, A. D., Lawlor, D. A., Sharara, F. I., & Nelson, S. M. (2020). *2008 financial crisis vs 2020 economic fallout: How COVID-19 might influence fertility treatment and live births*. Advance online publication. 10.1101/2020.10.18.20214650 10.1016/j.rbmo.2021.03.017 33931369

[CR25] Hale, T., Angrist, N., Goldszmidt, R., Kira, B., Petherick, A., Phillips, T., & Tatlow, H. (2021). A global panel database of pandemic policies (Oxford COVID-19 Government Response Tracker). *Nature Human Behaviour,**5*(4), 529–538. 10.1038/s41562-021-01079-8 10.1038/s41562-021-01079-8 33686204

[CR26] Hamilton, B. E., Martin, J. A., & Osterman, M. J. K. (2021). Births: Provisional data for 2020. *NVSS Vital Statistics Rapid Release,**012*, 1–12.

[CR27] Hellstrand, J., Nisén, J., Miranda, V., Fallesen, P., Dommermuth, L., & Myrskylä, M. (2021). Not just later, but fewer: Novel trends in cohort fertility in the Nordic countries. *Demography,**58*(4), 1373–1399. 10.1215/00703370-9373618 34251453 10.1215/00703370-9373618

[CR28] Hoffman, L. W., & Hoffman, M. L. (1973). The value of children to parents: A new approach to the study of fertility. In J. T. Fawcett (Ed.), *Psychological perspectives on population* (pp. 19–76). Basic Books.

[CR29] Huebener, M., Waights, S., Spiess, C. K., Siegel, N. A., & Wagner, G. G. (2021). Parental well-being in times of Covid-19 in Germany. *Review of Economics of the Household,**19*(1), 91–122. 10.1007/s11150-020-09529-4 33469413 10.1007/s11150-020-09529-4 PMC7808123

[CR30] Januszek, S. M., Faryniak-Zuzak, A., Barnaś, E., Łoziński, T., Góra, T., Siwiec, N., & Kluz, T. (2021). The approach of pregnant women to vaccination based on a COVID-19 systematic review. *Medicina*. 10.3390/medicina57090977 10.3390/medicina57090977 PMC8468958 34577900

[CR31] Jdanov, D., Sobotka, T., Zeman, K., Jasilioniene, A., Alustiza Galarza, A., Németh, L., & Winkler-Dworak, M. (2022). Short-Term Fertility Fluctuations Data series (STFF) – Methodological note (Human Fertility Database). Rostock, Vienna. Retrieved from https://www.humanfertility.org/Docs/STFFnote.pdf

[CR37] Kolk, M., Drefahl, S., Wallace, M., & Andersson, G. (2022). *Excess mortality and COVID-19 in Sweden in 2020: A demographic account*. Vienna Yearbook of Population Research.

[CR38] Kreyenfeld, M. (2016). Economic uncertainty and fertility. In K. Hank & M. Kreyenfeld (Eds.), *Kölner Zeitschrift für Soziologie und Sozialpsychologie Sonderheft. Social demography* (pp. 59–80). Springer.

[CR39] Lappegård, T., Kornstad, T., Dommermuth, L., & Kristensen, AP. (2022). Understanding the positive effects of the COVID-19 pandemic on women’s fertility in Norway: Discussion Paper No. 979. Statistisk sentralbyrå: Statistisk sentralbyrå. Retrieved from https://ssb.brage.unit.no/ssb-xmlui/handle/11250/2995390

[CR40] Ludwig-Walz, H., Dannheim, I., Pfadenhauer, L. M., Fegert, J. M., & Bujard, M. (2022). Increase of depression among children and adolescents after the onset of the COVID-19 pandemic in Europe: A systematic review and meta-analysis. *Child and Adolescent Psychiatry and Mental Health,**16*(1), 109. 10.1186/s13034-022-00546-y 36587221 10.1186/s13034-022-00546-y PMC9805372

[CR41] Ludwig-Walz, H., Dannheim, I., Pfadenhauer, L. M., Fegert, J. M., & Bujard, M. (2023). Anxiety increased among children and adolescents during pandemic-related school closures in Europe: A systematic review and meta-analysis. *Child and Adolescent Psychiatry and Mental Health,**17*(1), 74. 10.1186/s13034-023-00612-z 37344892 10.1186/s13034-023-00612-z PMC10286360

[CR42] Lundkvist, L. (2022). Third child – a new trend after Covid? Presentation to the 22nd Nordic Demographic Symposium in Oslo, June 9-11, Oslo

[CR43] Lundkvist, L. (2023). Personal communication, Oslo

[CR44] Matysiak, A., Sobotka, T., & Vignoli, D. (2021). The great recession and fertility in Europe: A sub-national analysis. *European Journal of Population,**37*(1), 29–64. 10.1007/s10680-020-09556-y 33597835 10.1007/s10680-020-09556-y PMC7864853

[CR45] Neyer, G., Andersson, G., Dahlberg, J., Ohlsson Wijk, S., Andersson, L., & Billingsley, S. (2022). Fertility decline, fertility reversal and changing childbearing considerations in Sweden: A turn to subjective imaginations? (Stockholm Research Reports in Demography)

[CR46] Nisén, J., Jalovaara, M., Rotkirch, A., & Gissler, M. (2022). Fertility recovery despite the COVID-19 pandemic in Finland? FLUX 4/2022 Working Papers; INVEST Working Papers 50/2022

[CR47] Ohlsson Wijk, S., & Andersson, G. (2023). Swedish fertility developments before, during and after the Covid-19 pandemic. Working Paper 10.1007/s10680-025-09744-8 PMC12279657 40691363

[CR48] Ohlsson-Wijk, S., & Andersson, G. (2022). Disentangling the Swedish fertility decline of the 2010s. *Demographic Research,**47*(12), 345–358. 10.4054/DemRes.2022.47.12

[CR49] Pötzsch, O. (2021). Geburtenknick oder Baby-Boom? Die Covid-19-Pandemie und die Geburtenentwicklung. Berliner Demografiegespräch, 2. November 2021, Berlin

[CR50] Public Health Agency of Sweden. (2022b). Statistik over registrerade vaccinationer covid-19. Retrieved from https://www.folkhalsomyndigheten.se/smittskydd-beredskap/utbrott/aktuella-utbrott/covid-19/statistik-och-analyser/statistik-over-registrerade-vaccinationer-covid-19/

[CR51] Public Health Agency of Sweden. (2022a). Information for people who are pregnant. Retrieved from https://www.folkhalsomyndigheten.se/publikationer-och-material/publikationsarkiv/i/information-about-vaccinations-for-people-who-are-pregnant/

[CR52] Public Health Agency of Sweden. (2023). När hände vad under pandemin? Logg on events in Sweden during the pandemic. Retrieved from https://www.folkhalsomyndigheten.se/smittskydd-beredskap/utbrott/aktuella-utbrott/covid-19/folkhalsomyndighetens-roll-under-arbetet-med-covid-19/nar-hande-vad-under-pandemin/#tjugoett

[CR53] Reid, A. (2005). The effects of the 1918–1919 influenza pandemic on infant and child health in Derbyshire. *Medical History,**49*(1), 29–54. 10.1017/s0025727300008279 15730129 10.1017/s0025727300008279 PMC1088249

[CR54] Ritchie, H., Mathieu, E., Rodés-Guirao, L., Appel, C., Giattino, C., Ortiz-Ospina, E., Roser, M. (2022). Coronavirus Pandemic (COVID-19). Retrieved from https://ourworldindata.org/coronavirus

[CR33] Robert-Koch-Institute. (2021a). Beschluss der STIKO zur 1. Aktualisierung der COVID-19-Impfempfehlung: 14. January 2021. Epidemiologisches Bulletin. (2)

[CR32] Robert-Koch-Institute. (2021b). COVID-19-Impfempfehlung der STIKO: Empfehlung für Schwangere und Stillende: 23. September 2021. Epidemiologisches Bulletin. (38)

[CR34] Robert-Koch-Institute. (2022a). COVID-19_Todesfälle nach Sterbedatum: Datenstand: 28.07.2022. Retrieved from https://www.rki.de/DE/Content/InfAZ/N/Neuartiges_Coronavirus/Projekte_RKI/COVID-19_Todesfaelle.html

[CR35] Robert-Koch-Institute. (2022b). Digitales Impfquoten-Monitoring COVID-19. Retrieved from https://www.rki.de/DE/Content/InfAZ/N/Neuartiges_Coronavirus/Daten/Impfquoten-Tab.html

[CR36] Robert-Koch-Institute. (2023). 7-Tage-Inzidenzen nach Bundesländern und Kreisen (fixierte Werte): Stand: 10.4.2023. Retrieved from https://www.rki.de/DE/Content/InfAZ/N/Neuartiges_Coronavirus/Daten/Inzidenz-Tabellen.html

[CR55] Sobotka, T., Jasilioniene, A., Galarza, A. A., Zeman, K., Németh, L., & Jdanov, D. (2021). Baby bust in the wake of the COVID-19 pandemic? First results from the new STFF data series. SocArXiv Papers. Advance online publication. 10.31235/osf.io/mvy62

[CR56] Sobotka, T., Zeman, K., Jasilioniene, A., Winkler-Dworak, M., Brzozowska, Z., Alustiza-Galarza, A., Jdanov, D. (2023). Pandemic roller-coaster? Birth trends in higher-income countries during the COVID-19 pandemic. *Population and Development Review*. Advance online publication. 10.1111/padr.12544

[CR57] Statistics Sweden. (2022). Statistikdatabasen: Labour Force Surveys. Retrieved from https://www.scb.se/en/finding-statistics/statistics-by-subject-area/labour-market/labour-force-surveys/labour-force-surveys-lfs/

[CR58] Statistics Sweden. (2023). Statistikdatabasen. Retrieved from https://www.statistikdatabasen.scb.se/pxweb/sv/ssd/START__BE__BE0101__BE0101G/ManadBefStatRegion/

[CR60] Statistisches Bundesamt. (2023a). Bevölkerung: Deutschland, Stichtag, Altersjahre, Geschlecht: Fortschreibung des Bevölkerungsstandes. Wiesbaden

[CR61] Statistisches Bundesamt. (2023b). Lebendgeborene nach Monaten - vorläufige Ergebnisse (Genesis No. 12411–0008). Wiesbaden

[CR59] Statistisches Bundesamt. (2023c). Statistik der Geburten: Lebendgeborene: Deutschland, Monate, Geschlecht (No. 12612–02). Wiesbaden

[CR62] Swedish National Board of Health and Welfare. (2022). Statistik om covid-19. Retrieved from https://www.socialstyrelsen.se/statistik-och-data/statistik/statistik-om-covid-19/

[CR63] Szabo, T. G., Richling, S., Embry, D. D., Biglan, A., & Wilson, K. G. (2020). From helpless to hero: Promoting values-based behavior and positive family interaction in the midst of COVID-19. *Behavior Analysis in Practice,**13*(3), 568–576. 10.1007/s40617-020-00431-0 32328219 10.1007/s40617-020-00431-0 PMC7178922

[CR64] Tavares, L. P., Azevedo, A. B., & Arpino, B. (2022). Fertility, economic uncertainty and the Covid-19 pandemic: Before and after. SocArXiv, 11 May 2022. Advance online publication. 10.31235/osf.io/n3cw8

[CR65] Vignoli, D., Guetto, R., Bazzani, G., Pirani, E., & Minello, A. (2020). A reflection on economic uncertainty and fertility in Europe: The narrative framework. *Genus,**76*(1), 28. 10.1186/s41118-020-00094-3 32921800 10.1186/s41118-020-00094-3 PMC7480209

[CR66] Wagner, S., Tropf, F. C., Cavalli, N., & Mills, M. C. (2020). Pandemics, public health interventions and fertility: Evidence from the 1918 influenza. SocArXiv, 24 Nov. 2020. Advance online publication. 10.31235/osf.io/f3hv8

